# Plethora of Non-Covalent Interactions in Coordination and Organometallic Chemistry Are Modern Smart Tool for Materials Science, Catalysis, and Drugs Design

**DOI:** 10.3390/ijms232314767

**Published:** 2022-11-25

**Authors:** Alexander S. Novikov

**Affiliations:** 1 Institute of Chemistry, Saint Petersburg State University, Universitetskaya nab. 7-9, 199034 Saint Petersburg, Russia; a.s.novikov@spbu.ru or novikov@itmo.ru; 2 Infochemistry Scientific Center, ITMO University, Lomonosova st. 9, 191002 Saint Petersburg, Russia

**Keywords:** non-covalent interactions, supramolecular systems, coordination chemistry, organometallic chemistry, material science, machine learning, artificial intelligence, computer modeling

## Abstract

Non-covalent interactions are one of the key topics in coordination and organometallic chemistry. Examples of such weak interactions are hydrogen, halogen, and chalcogen bonds, stacking interactions, metallophilic contacts, etc. Non-covalent interactions play an important role in materials science, catalysis, and medicinal chemistry. The aim of this Special Issue of *International Journal of Molecular Sciences*, entitled “Non-Covalent Interactions in Coordination and Organometallic Chemistry”, is to cover the most recent progress in the rapidly growing field of non-covalent interactions in coordination and organometallic chemistry. Both experimental and theoretical studies, fundamental and applied research and any types of manuscripts are welcome for consideration.

Non-covalent interactions are one of the key topics in coordination and organometallic chemistry. Examples of such weak interactions are hydrogen, halogen, and chalcogen bonds, stacking interactions, metallophilic contacts, etc. Non-covalent interactions control the structure of solids (mainly molecular crystals), as well as the properties of supramolecular systems in liquid and gas phases, in addition, the elementary stages of chemical reactions are due to such weak inter- and intramolecular contacts. In the context of bio(organic/inorganic)chemistry, non-covalent interactions are necessary for the formation and folding of the three-dimensional structure of proteins and nucleic acids as well as their metal complexes and ligand-protein binding. These inter- and intramolecular interactions have a significant effect on the properties of polymers, gels and membranes, on the dissolving abilities of liquids and their boiling points. Non-covalent interactions play an important role in materials science, catalysis, and medicinal chemistry. Information about inter- and intramolecular interactions can be obtained experimentally, for example, based on X-ray diffraction analysis data, from phonon spectra and elastic characteristics of crystals, using thermogravimetric analysis and measurements of sublimation energies, from spectral characteristics (for example, UV-Vis and IR spectroscopy, Raman spectroscopy, NMR spectroscopy). At the same time, these experimental methods provide only indirect information, largely mediated by the experimental conditions, and often do not allow one to establish the nature (in particular, answer the question: are certain considered non-covalent contacts binding or repulsive?) and the energy of non-covalent interactions, what can be the goal and task for computer modeling and computational chemistry. In particular, the most promising theoretical methods in this context are the topological analysis of the electron density distribution within the formalism of Bader’s theory (QTAIM) using additional auxiliary methods: visualization of van der Waals potential, Interaction Region Indicator (IRI) analysis, Independent Gradient Model (IGM) analysis, Density Overlap Regions Indicator (DORI) analysis, localized orbital locator (LOL) analysis, electron localization function (ELF) analysis, reduced density gradient (RDG) analysis, etc.); visualization and mapping of non-covalent interactions (for example, using Noncovalent Interaction (NCI) analysis) and the study of the degree of influence of crystal packing on the structure of isolated molecular associates stabilized by non-covalent contacts using Hirshfeld surface analysis; analysis of orbitals and charges, in particular, within the formalism of Weinhold’s theory (NBO); study of energy and charge decomposition within the formalism of EDA and CDA theories, respectively. Thus, the most adequate, comprehensive, and promising approach to the study of non-covalent interactions in chemical compounds and their supramolecular associates seems to be a combination of experimental observations and computer simulation results using the latest advances in quantum and computational chemistry. Finally, processing of large amounts of data (quantitative indicators of properties and energy characteristics of various supramolecular contacts in coordination and organometallic compounds), machine learning and artificial intelligence (building predictive models using mathematical statistics, numerical methods, mathematical analysis, optimization methods, probability theory, graph theory, various techniques for working with data in digital form) is very promising goal for data scientists.

The aim of this Special Issue of *International Journal of Molecular Sciences*, entitled “Non-Covalent Interactions in Coordination and Organometallic Chemistry”, is to cover the most recent progress in the rapidly growing field of non-covalent interactions in coordination and organometallic chemistry. Both experimental and theoretical studies, fundamental and applied research and any types of manuscripts are welcome for consideration.

This Special Issue will address the following topics:

- experimental studies of non-covalent interactions;
- theoretical modeling of supramolecular systems;
- 1D, 2D, 3D coordination polymers;
- weak contacts in bio(organic/inorganic)chemistry;
- non-covalent interactions in material science;
- application of machine learning and artificial intelligence in studies of non-covalent interactions.

We welcome researchers to contribute their works to our Special Issue. We hope that the materials of this Special Issue will prove valuable and useful to a wide range of readers and researchers and will serve as a starting point for new fruitful work in the field of non-covalent interactions in coordination and organometallic chemistry. All types of manuscripts (reviews, mini-reviews, full papers, short communications, technical notes, highlights, etc.) are welcome for consideration.

## Data Availability

Not applicable.

